# Does Femoral Arterial Calcification Have an Effect on Mortality in Patients Who Underwent Hemiarthroplasty Due to Hip Fracture?

**DOI:** 10.7759/cureus.46437

**Published:** 2023-10-03

**Authors:** Hakan Yolaçan, Serkan Güler

**Affiliations:** 1 Orthopaedics and Traumatology, Aksaray University Training and Research Hospital, Aksaray, TUR

**Keywords:** calcification, hemiarthroplasty, mortality, femoral arterial calcification, hip fracture

## Abstract

Aim: We aimed to investigate the effect of femoral arterial calcification on mortality in patients who underwent hemiarthroplasty due to hip fracture.

Material and methods: In our study, 481 patients who were operated for hip fracture between 01.01.2015 and 01.01.2021 were evaluated retrospectively. Femoral arterial calcification on the fractured side was evaluated in the preoperative pelvic anteroposterior (AP) X-ray, and the patients were divided into two subgroups according to the presence or absence of femoral arterial calcification. The overall survival and first-month and first-year survival of the patients were evaluated. Patients' age, gender, side, fracture type, treatment method, time between fracture and operation date, presence of femoral arterial calcification and type of anesthesia (regional, general) were recorded.

Results: Of the 481 patients included in the study, 299 were female and 182 were male, and the mean age was calculated as 80.5. Of the patients, 187 were diagnosed with femoral neck fractures and the remaining 294 with pertrochanteric fractures. It was observed that the mortality rate in the first month after surgery was 58 (12%) for both groups, and the mortality rate in the first year was 173 (35.9%) for both groups. The overall postoperative mortality was calculated as 302 (62.7%) for both groups. Femoral arterial calcification was detected in 191 of 481 patients, and femoral arterial calcification was not observed in the remaining 290 patients. Similarly, when both groups were compared in terms of mortality in the first month after surgery, mortality in the first year and overall mortality rates, no significant difference was found between the groups (p>0.05).

Conclusion: In our study we showed that femoral arterial calcification has no effect on mortality in acute hip fractures treated by hemiarthroplasty in people over 65 years of age.

## Introduction

Many changes occur in the musculoskeletal system with aging, and the most important one is the loss of muscle and bone density, which causes changes in the geometry of the bone. Depending on these changes, there is also a decrease in the coordination and speed of movements, and a decrease in the strength of the movements and the bone load carrying capacity are observed [1]. It is estimated that there will be more than six million hip fracture cases in 2050 due to the increase in life expectancy [2,3]. Mortality for hip fractures can be as high as 30% in the six-month period and up to 50% in the one-year period [4,5]. Mortality rate after hip fracture is related to many clinical factors such as age, gender, and time from fracture to surgery [6-8].

Arterial calcification is an independent predictor of mortality and morbidity with increasing age [9]. Arterial calcification in the lower extremity doubles cardiovascular mortality [10]. It is divided into three types: intimal type, medial artery calcification (MAC) and mixed type. Intimal-type calcification is a marker of atherosclerotic disease and is associated with arterial stenosis. In MAC-type calcification, the elastic layer of the arterial wall is affected. In the mixed type, both intimal and medial calcifications are seen in the arterial wall [11].

Arterial calcification causes osteoporosis by disrupting the physiological dynamic processes of vessels and bones, resulting in an increase in bone fragility [12]. For example, the presence of aortic calcification is an independent factor causing osteoporosis in the proximal femur [13].

In this study, we aimed to investigate the effect of femoral artery calcification (FAC) on mortality in patients who underwent hemiarthroplasty due to hip fracture.

The hypothesis of the study is that the presence of FAC increases mortality in elderly patients who underwent surgery for hip fracture.

## Materials and methods

In our study, 481 patients who were operated for hip fracture between 01.01.2015 and 01.01.2021 were evaluated retrospectively. This study was approved by the Ethics Committee of Aksaray University Training and Research Hospital (Approval No:2022/14-03). There is no information about patients in the submitted manuscript. The population of the study is patients over 65 years of age with acute hip fracture. Patients over 65 years of age who were operated on with the diagnosis of acute hip fracture (femur neck fracture, pertrochanteric fracture) and who survived the operation, with a minimum follow-up period of one year, and fractures after low-energy trauma were included in the study. Pathological fractures, periprosthetic fractures, fractures after high-energy trauma, patients with a history of multiple trauma, and those treated conservatively were not included in the study.

**Figure 1:**
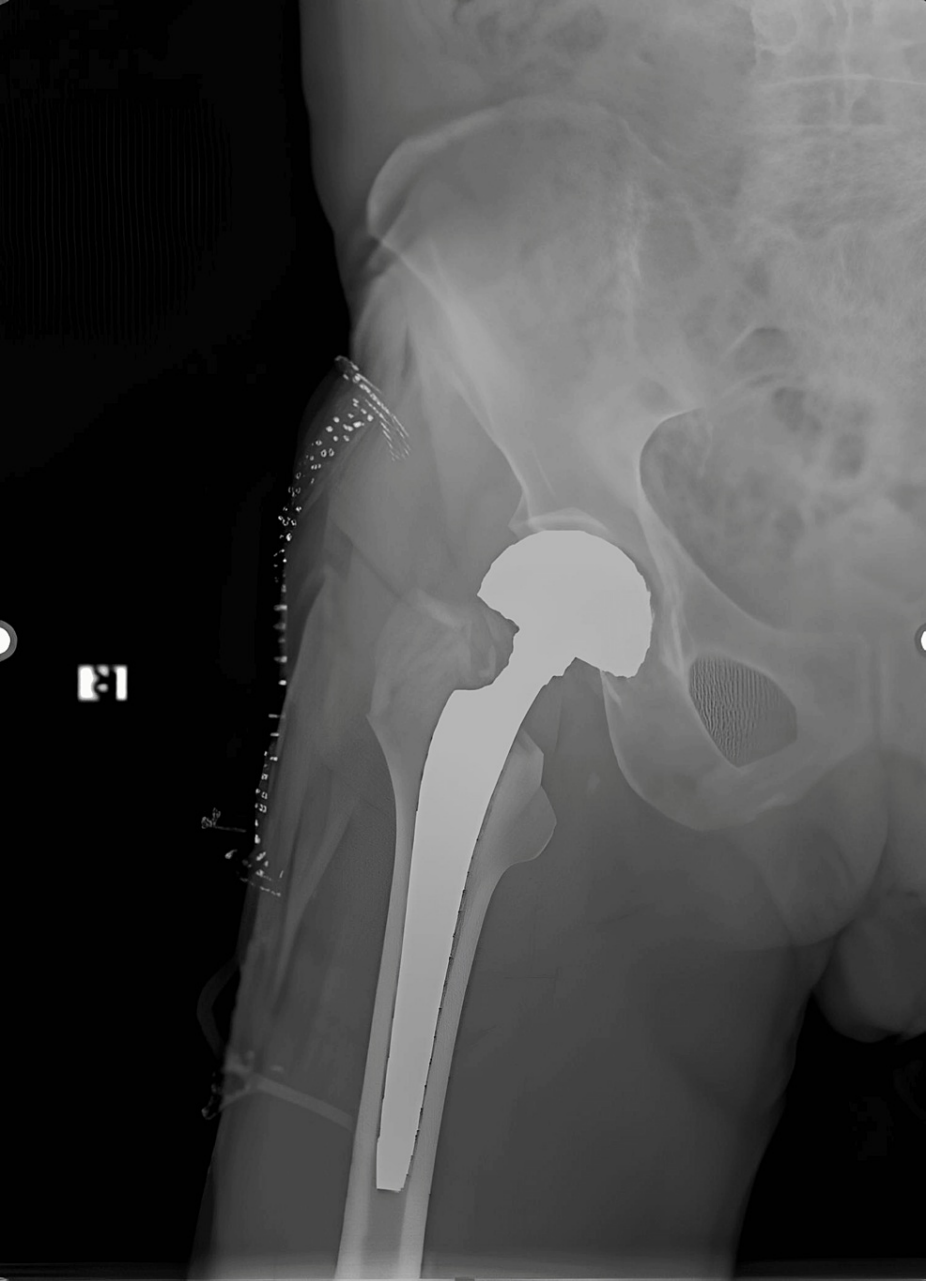
Postoperative X-ray image of femoral neck fracture treated with hemiarthroplasty

In our clinic, weight-bearing mobilization is routinely started on the first postoperative day for patients undergoing hemiarthroplasty, and the Kocher-Langenback approach is used as the standard for surgical incision in patients. Additionally, uncemented surgical technique is preferred. Figure 1 shows the postoperative X-ray image of a patient who underwent hemiarthroplasty.

**Figure 2:**
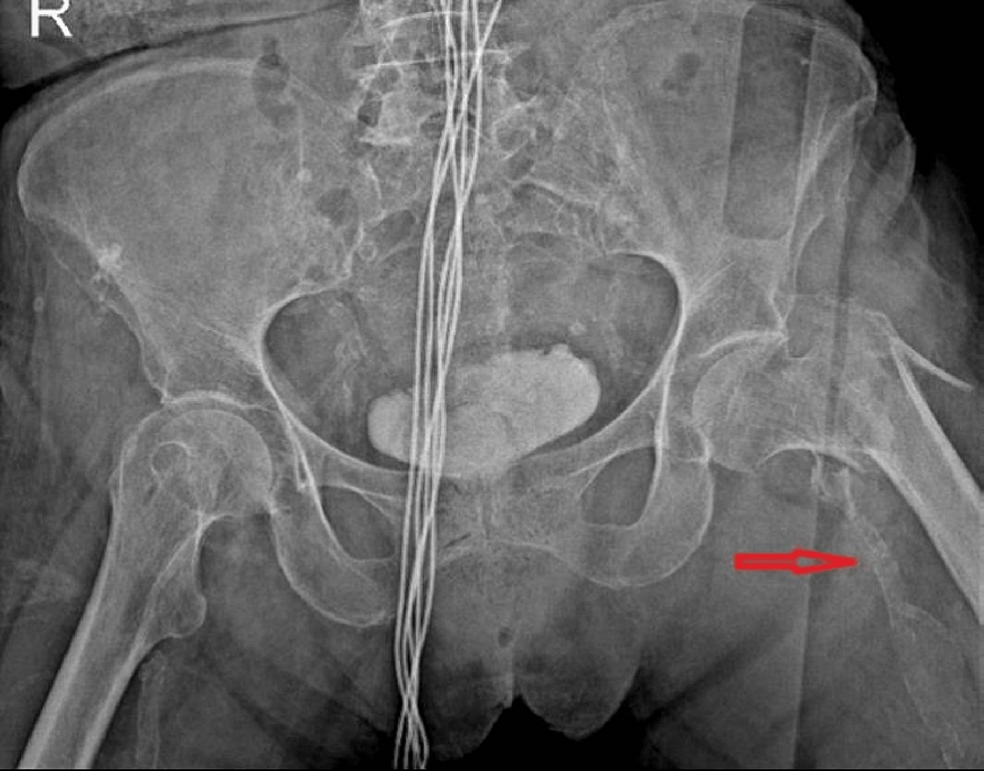
Radiological image of the femoral artery calcification on the fractured side in pelvis anteroposterior (AP) X-ray

The presence of FAC was evaluated on the fractured side on the preoperative pelvic anteroposterior (AP) X-ray, and the patients were divided into two groups according to the presence of FAC (Figure 2).

In order to determine the death status and date of death of the patients, data were collected from the Death Information System affiliated with the General Directorate of Health Systems in our country.

The overall survival and first-month and first-year survival of the patients were evaluated. Patients' age, gender, side, fracture type, treatment method, time between fracture and operation date, presence of FAC and type of anesthesia (regional, general) were recorded.

Statistical analysis

In our study, the data of the patients who were operated on for hip fracture and who met the inclusion and exclusion criteria were analyzed using SPSS version 26 (IBM Corp., Armonk, NY, USA). The homogeneity of the data was evaluated with the Kolmogorov-Smirnov test. Student's t-test was used to compare data. A p-value of less than 0.05 was accepted as statistically significant.

## Results

Of the 481 patients included in the study, 299 were female and 182 were male, and the mean age was calculated as 80.5. Of the patients, 187 were diagnosed with femoral neck fractures and the remaining 294 with pertrochanteric fractures. Since it was seen that all of the patients were treated with hemiarthroplasty, it was not considered a separate variable and was not included as a criterion in the study. It was determined that 472 of the patients were given regional anesthesia and nine of them were given general anesthesia. The mean follow-up period of the patients was calculated as 51.3 months. It was observed that 58 patients (12%) died in the first month after the operation, and 173 patients (35.9%) died one year later. In all follow-ups, it was observed that 302 patients (62.7%) died after surgery.

FAC was detected in 191 of 481 patients, and FAC was not observed in the remaining 290 patients. When the groups with and without FAC were compared in terms of age, gender, side, fracture type, anesthesia type, and time to surgery, no significant difference was found between the groups (p>0.05) (Table 1). Similarly, when both groups were compared in terms of mortality in the first month after surgery, mortality in the first year and overall mortality rates, no significant difference was found between the groups (p>0.05) (Table 1).

**Table 1:** Comparison of demographic data, mortality and clinical information of both groups FAC: Femoral arterial calcification, F: Female, M: Male, R: Right, L: Left, C: Collum femoris fracture, P: Pertrochanteric fracture, Rg: Regional anesthesia, Gen: General anesthesia

Patient data	Patients with FAC (N=191)	Patients without FAC (N=290)	p-value
Age, years	80.1±10.1	80.7±8.3	0.45
Gender, F/M	109/82	190/100	0.063
Side, R/L	85/106	128/162	0.937
Type of fracture, C/P	73/118	114/176	0.992
Time to surgery, day	1.71±0.48	1.73±0.51	0.627
Type of anesthesia, Rg/Gen	188/3	284/6	0.694
Mortality, in first month	24 (12.5%)	34 (11.7%)	0.782
Mortality, in first year	67 (35%)	106 (36.5%)	0.742
Mortality, overall	116 (60.7%)	186 (64.1%)	0.451
Follow-up time, months	52.4	50.6	0.985

## Discussion

The most important finding of our study is that FAC has no effect on postoperative first-month and first-year mortality and general mortality in patients who underwent hemiarthroplasty due to hip fracture.

Many factors affect mortality and outcomes after hip fracture. In addition to factors such as age, gender, ASA score, operation time, and additional diseases, it has been shown that cemented or uncemented application in patients undergoing hemiarthroplasty, and different nail types if proximal femoral nail (PFN) is applied in intertrochanteric fractures, affect the results [14-16].

Most of the studies on structures related to hip fracture have investigated the factors affecting mortality. Previous studies have shown that arterial calcification has an active and complex process and accordingly, impairs bone-associated protein synthesis [17,18]. The effect of vascular calcification on the bone is osteoporosis and it causes fractures due to the deterioration in the bone formation and destruction cycle [12].

Moreover, it is accepted that lower extremity vascular calcification is an independent and important factor for cardiovascular events [19]. Similarly, Chowdhury et al. showed that arterial calcification in the lower extremity is associated with mortality [20]. In another study, it was shown that the amount of plaque in the femoral artery was associated with higher loss of motion and immobilization [21]. Rennenberg et al. in a meta-analysis study conducted by 218,080 patients with an average follow-up of 10.1 years stated that the presence of calcification in any artery wall was associated with three to four times higher mortality and this condition occurs in the general population and is not limited to patients undergoing surgery [9]. Huang et al. in a study of 82 patients with symptomatic peripheral artery disease showed that disease severity was associated with lower extremity artery calcification and the mortality rate due to all causes was higher [16]. In the study performed by Birişik et al., 540 hips of 530 patients were examined and it was shown that the one-month, one-year and all mortality rates after surgery were higher in the group with FAC [22]. In another study conducted by Bayram et al., 304 patients were evaluated, and similarly, it was reported that mortality rates were significantly higher in the group with FAC [23].

On the other hand, in Pazarcı et al., 145 patients over 65 years of age who were operated for hip fracture were examined it was shown that FAC had no effect on mortality [24].

In our study, it was shown that FAC had no effect on first-month, first-year and all-time mortality rates in acute hip fracture patients over 65 years of age. When the studies that concluded to have an effect on mortality and our study were compared, we thought that the higher number of patients who underwent the same type of surgery in our study may cause the difference. The number of our patients was determined by Pazarcı et al. It was significant in terms of our study that it was more than the study conducted by them and the results were similar.

Study limitations

Limitations of our study are the retrospective nature, not evaluating the operation time, not including additional diseases, and not investigating different treatment methods due to the application of a single type of surgery (hemiarthroplasty). Additionally, regression analysis was not used.

## Conclusions

We showed that femoral arterial calcification had no effect on mortality in patients over 65 years of age who underwent hemiarthroplasty for acute hip fracture.
